# Patient centred variables with univariate associations with unplanned ICU admission: a systematic review

**DOI:** 10.1186/s12911-019-0820-1

**Published:** 2019-05-15

**Authors:** James Malycha, Timothy Bonnici, David A. Clifton, Guy Ludbrook, J. Duncan Young, Peter J. Watkinson

**Affiliations:** 1 Kadoorie Centre for Critical Care Research and Education, Nuffield Department of Clinical Neurosciences, University of Oxford, Level 3, John Radcliffe Hospital, Headley Way, Oxford, OX3 9DU UK; 2 Department of Critical Care, University College London Hospitals Foundation Trust, Maple Link Bridge, University College Hospital, 235 Euston Road, London, NW1 2BU UK; 3 Department of Engineering Science, Institute of Biomedical Engineering, University of Oxford, Old Road Campus, Roosevelt Drive, Oxford, OX3 7DC UK; 4 Faculty of Health and Medical Science, University of Adelaide, North Terrace, AHMS Floor 8, Adelaide, 5000 Australia

**Keywords:** Critical care, Intensive care, ICU admission, Clinical deterioration, EPR, EHR, Variable selection, Systematic review, Predictive scores

## Abstract

**Background:**

Multiple predictive scores using Electronic Patient Record data have been developed for hospitalised patients at risk of clinical deterioration. Methods used to select patient centred variables for inclusion in these scores varies. We performed a systematic review to describe univariate associations with unplanned Intensive Care Unit (ICU) admission with the aim of assisting model development for future scores that predict clinical deterioration.

**Methods:**

Data sources were MEDLINE, EMBASE, CINAHL, CENTRAL and the Cochrane Database of Systematic Reviews. Included studies were published since 2000 describing an association between patient centred variables and unplanned ICU admission determined using univariate analysis. Two authors independently screened titles, abstracts and full texts against inclusion and exclusion criteria. DistillerSR (Evidence Partners, Canada, Ottawa, Ontario) software was used to manage the data and identify duplicate search results. All screening and data extraction forms were implemented within DistillerSR. Study quality was assessed using an adapted version of the Newcastle-Ottawa Scale. Variables were analysed for strength of association with unplanned ICU admission.

**Results:**

The database search yielded 1520 unique studies; 1462 were removed after title and abstract review; 57 underwent full text screening; 16 studies were included. One hundred and eighty nine variables with an evaluated univariate association with unplanned ICU admission were described.

**Discussion:**

Being male, increasing age, a history of congestive cardiac failure or diabetes, a diagnosis of hepatic disease or having abnormal vital signs were all strongly associated with ICU admission.

**Conclusion:**

These findings will assist variable selection during the development of future models predicting unplanned ICU admission.

**Trial registration:**

This study is a component of a larger body of work registered in the ISRCTN registry (ISRCTN12518261).

**Electronic supplementary material:**

The online version of this article (10.1186/s12911-019-0820-1) contains supplementary material, which is available to authorized users.

## Background

In experimental settings, scores that predict risk for clinical deterioration in hospitalised patients have evolved from vital sign based Early Warning Scores (EWS) to systems that utilise the large amount of patient centred data in Electronic Patient Records (EPRs) [1–4]. These systems are not yet in widespread use, however they represent a first step towards *automatically* assimilating patient data to assist clinical decision making on high risk ward patients. Each of the current, published experimental models were derived and validated on large EPR linked databases that used Intensive Care Unit (ICU) admission as one of the outcome measures. This outcome measure is commonly used (along with death and cardiac arrest) as a surrogate for confirmed clinical deterioration.

These and other prognostic models use a variety of statistical methods but multivariate regression modelling and machine learning techniques are commonly used. These methods require patient centred ‘candidate’ variables (such as vital signs or laboratory results) to form the component parts of the model [5]. The process of selecting model candidate variables is important, however there is no consensus on how best to do this. Numerous methods have been used for multivariate logistic regression, including expert opinion, forward and backward stepwise selection and machine learning techniques [6]. A logical and often used first step is evaluating univariate associations, which enables the variables to be quantified in advance of their inclusion in the model [7]. This is helpful when using EPR data where there are large number of available candidate variables [8]. Regardless of the method, the goal is to include the *optimal* combination of variables that maximise predictive ability, whilst avoiding unnecessary complexity [6].

In this systematic review we provide a complete summary of patient centred variables with a *univariate* association with unplanned ICU admission. By providing these data, we hope to aid the development of EPR based models for the prediction of ICU admission (and therefore clinical deterioration). We anticipate these data will enhance data-driven improvements in the care of deteriorating ward patients.

## Methods

### Search and identification of studies

The study protocol has been published [9] and follows the Preferred Reporting of Observational Studies and Meta-Analysis (PRISMA) statement [10]. An experienced medical librarian helped devise the search strategy to maximise identification of relevant studies. Studies were identified by searching Medical Literature Analysis and Retrieval System Online (MEDLINE), Excerpta Medica database (EMBASE), Cumulative Index to Nursing and Allied Health Literature (CINAHL), the Cochrane Database of Systematic Reviews and the Cochrane Central Register of controlled trials (CENTRAL). We included additional studies from the references of review articles, studies identified during screening, and from the authors’ personal libraries. We restricted the search to studies published since 2000. We did not apply any language restrictions. The search design is shown in the Additional file 1 (SDC-1).

### Inclusion criteria

Included studies evaluated hospitalised, adult patients located in either the Emergency Department (ED), the general surgical or medical wards. Patients in specialist wards (such as obstetric or psychiatric) were eligible if they were evaluated as a part of the general patient population rather than disease specific sub groups of patients. Included studies required an analysis of at least two cohorts: one cohort of patients admitted to ICU (intervention) and one not admitted to ICU (control). Variables were eligible if they were patient centred and had been evaluated across both cohorts. Studies which described a univariate, statistical relationship between a patient centred variable (e.g. heart rate) and unplanned ICU admission were included. The described variables were single entities, as opposed to composites such as risk scores.

### Exclusion criteria

Excluded studies did not evaluate unplanned ICU as an isolated outcome measure nor did they evaluate patients requiring ICU readmission. Additionally, studies that evaluated variables related to hospital processes or environmental risk alone (e.g. staff-to-nurse ratios), carried out multivariate analyses (without describing the univariate analyses that went into selecting variables for the model) or evaluated patient groups with a single diagnosis, were also excluded. (The studies excluded via this criterion are listed in Additional file 1 (SDC-2)). Patients admitted to ICU (or not) from high acuity areas such as HDU were excluded from the review as these hospital areas are often linked to ICUs and so are not always captured as admissions. Subgroups of illness acuity, such as needing an Rapid Response System (RRS) alert or being a high triage category, were not excluded. Finally, studies not published in peer-reviewed journals and those examining patients < 15 years old were excluded.

### Study selection and data abstraction

**Fig. 1:**
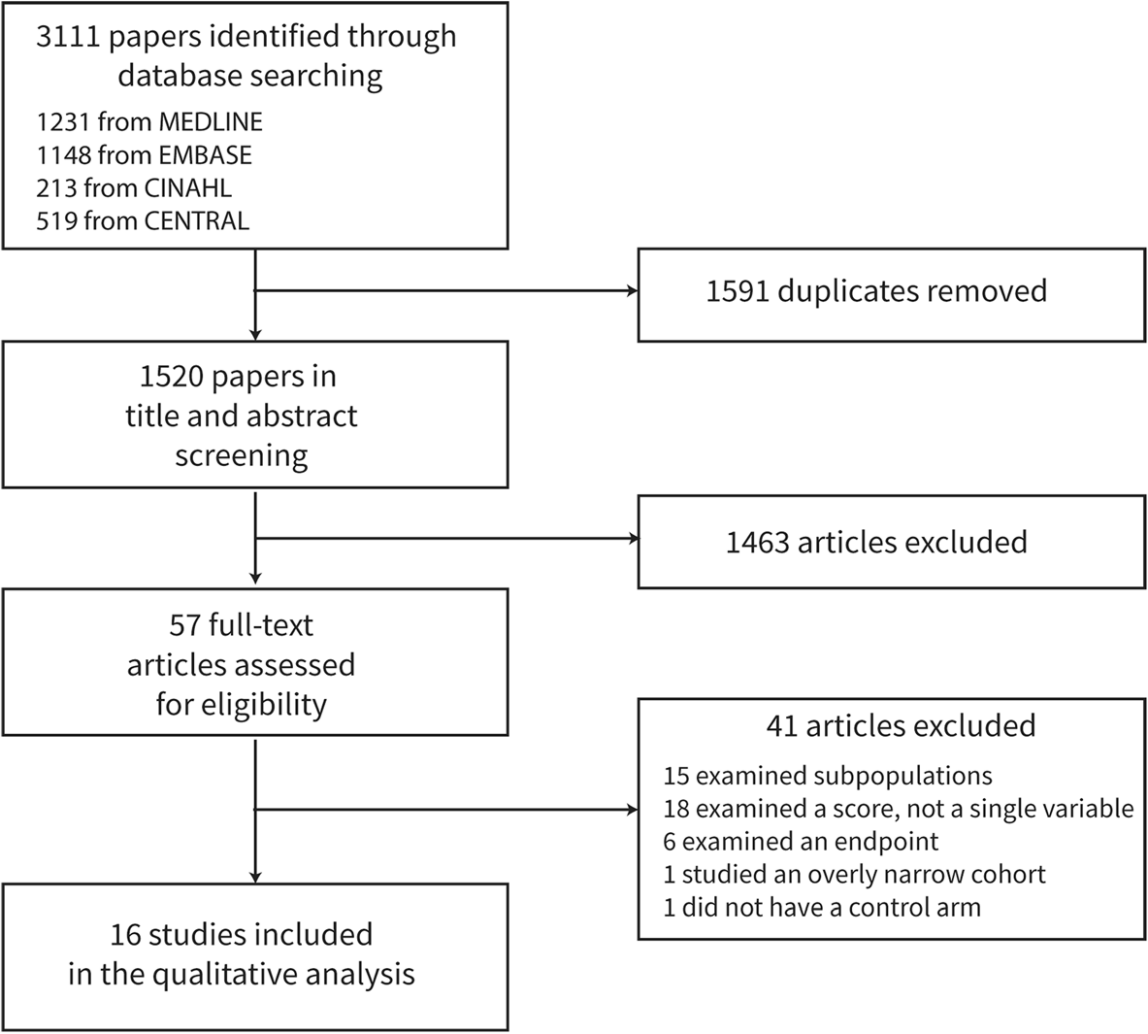
Flow diagram of the included and excluded studies

Two authors (JM, TB) independently screened titles and abstracts of identified studies against the inclusion and exclusion criteria. (Fig. 1) They were not blinded to the journal titles or to the study authors or institutions. If there was disagreement or uncertainty regarding eligibility, the article was included in the next stage of screening. The full text was retrieved for all articles not excluded by the initial screening and re-assessed for eligibility as before. Disagreements about eligibility were resolved by discussion between the screening authors or a third party (a senior member of our research team, PW and DY). Two authors extracted independently data from the studies and supplementary material. Any uncertainties regarding data extraction were resolved by discussion amongst the study team. DistillerSR (Evidence Partners, Canada, Ottawa, Ontario) was used to manage the data and identify duplicate search results. All screening and data extraction forms were implemented within DistillerSR.

### Risk of Bias assessment

Two authors (JM, TB) independently assessed the risk of bias for included studies by using an adapted version of the Newcastle-Ottawa Scale (NOS) [11, 12]. The NOS is a scoring system designed to assess the quality of non-randomised studies in meta-analyses and systematic reviews [13]. We generated a score by assessing each study cohort for representativeness of the desired study population, the comparability of the cohorts being assessed, the size of the cohorts and correction for confounding. We adapted the NOS score to show bias in the types of studies included in this review (i.e. those showing univariate associations) whilst remaining faithful to the established NOS methodology. The details of the modified NOS scale are included in the Additional file 1 (SDC-3).

### Data synthesis

We categorised patient-derived variables as comorbidities, demographics, laboratory tests, vital signs, diagnoses, medications and symptoms/signs, in general accordance with the categories used in the included studies. To synthesise and present the large number of variables included in the results in a logical way, we adopted the recently published method of Dettmer et al. (as adopted from Zaal et al.), who combined the quality of the studies investigating the variables in question (based on the NOS risk of bias assessment) with the number of times the variable was investigated [14, 15]. This semi-quantitative approach enable the assignation of a ‘weight of evidence’ to each variable (Table 1).

**Table 1:** Grading system for strength of evidence

Strength of Evidence	Criteria
Strong	≥2 high-quality studies showing positive association between the presence of a variable and the outcome AND No studies showing a negative association
Moderate	One high-quality AND one lesser-quality study showing association AND No studies showing negative association
Weak	> 2 low-quality studies showing positive association OR Only one high-quality study showing positive association
Negative	≥ 1 high-quality study showing negative association (inverse relationship) AND No studies showing a positive association
Inconclusive	Associations present in only one low-quality study OR No studies of any quality showing univariate association OR Presence of positive and negative associations from different articles, regardless of study quality

### RESULTS

All included studies are shown in Table 2 [16–31], The database search yielded 1520 unique studies; 1462 were removed after title and abstract review; 57 underwent full text screening; sixteen studies were included in this review (Fig. 1). Summary details are shown in Table 2 with additional study data in the Additional file 1 (SDC-4). The mean study quality score was five and the mode was seven. We graded six studies high quality [18, 20, 21, 28–30], four moderate quality [16, 23–25] and six (low quality) [17, 19, 22, 26, 27, 31]. The results of the bias assessment for each study are shown in Table 2 and the Additional file 1 (SDC-5). The quality of the studies is also reflected in the weight of evidence for any particular variable.

**Table 2:** Details of included studies

Ref	Lead Author	Publication year	Total number of patients in study	Patients in ICU group	Country	No of sites	Bias scores (high (HQ), medium (MQ) or low quality (LQ))
[16]	Barfod	2012	6279	102	Denmark	1	5 - MQ
[17]	Calzavacca	2012	95	15	Australia	1	2 - LQ
[18]	Churpek	2013	59,643	2638	USA	1	7 - HQ
[19]	Eick	2015	5730	366	Germany	1	4 - LQ
[20]	Escobar	2012	102,488	3525	USA	14	7 - HQ
[21]	Frost	2009	126,826	1582	Australia	1	7 - HQ
[22]	Hong	2011	1025	201	Singapore	1	4 - LQ
[23]	Hunziker	2012	74,784	5233	USA	1	5 - MQ
[24]	Loekiko	2013	70,829	149	Australia	2	5 - MQ
[25]	Schuetz	2015	7000	490	Swiss, France, USA	3	5 - MQ
[26]	Steiner	2016	2407	93	Switzerland	1	4 - LQ
[27]	Sudarshan	2015	527	42	USA	1	3 - LQ
[28]	Tam	2008	94,482	672	Australia	1	7 - HQ
[29]	Tsai	2014	1049	313	Taiwan	1	7 - HQ
[30]	Tsai	2014	699	214	Taiwan	1	7 - HQ
[31]	Wunderink	2012	214	71	USA	1	3 - LQ

Quantised data were presented as independent variables. For example, arterial oxygen saturation was evaluated six times for ranges of < 80, < 95%, 80–89%, 90–94%, mean (%) and median (%) (in each study group) across four studies and thus included six times in the initial analysis, with each of these ranges being defined as a single variable [16, 18, 20, 22]. Likewise, ‘cardiovascular disorder’ was included 14 times across six studies, as either a comorbidity, diagnosis or symptom/sign [21, 26–29]. We recorded the statistical relationship between variables and unplanned ICU admission as *p* values, Odds Ratios (OR), Risk Ratios (RR) [28] or Incidence Rate Ratios (IRR) [21] (with 95% confidence intervals) and did not assign preference.

Five studies (31%) were case control studies and 11 (69%) were cohort studies. Eight (50%) were prospective and eight (50%) were retrospective. The number of participants in each study ranged between 95 in a prospective cohort study and 126,826 in a retrospective case control study. The number of patients admitted to ICU ranged between 15 and 5233, while in the control group they ranged between 80 and 125,244 (Table 2). Five studies (31%) evaluated patients in emergency departments (ED) and 11 studies (69%) evaluated patients treated on hospital wards. Of the studies examining ward patients, five evaluated patients admitted via ED, two evaluated patients who had an RRS review and four evaluated patients admitted via any source (SDC-6, Additional file 1). Escobar [20] studied patients in 20 centres, Schuetz [25] in three centres and Loekiko [24] in two centres. The remaining 13 studies were single centre (Table 1).

Across the 16 studies, 189 different patient-derived variables were assessed for univariate association with unplanned ICU admission. Of these, 53 were vital signs, 42 were comorbidities, 29 were diagnosis, 26 were demographics, 25 were laboratory results, 10 were symptoms/signs and 4 were medications. One hundred and twenty-eight variables had a statistically significant positive association, two had a negative association and 59 had no association with unplanned ICU admission. Information on effect size was described as ORs, RRs or IRRs where available and is shown in Additional file 1 (SCD-7).

The semi-quantitative analysis resulted in 110 variables after repeatedly measured variables were grouped together. These are shown in Table 3 and Additional file 1 (SDC-8). Overall there were 12 variables with a strong weight of evidence (one was negative), three with a moderate weight of evidence and 33 with a weak weight of evidence for an association with unplanned ICU admission. The remaining 62 variables showed an inconclusive weight of evidence.

**Table 3:** Patient centred variables associated with unplanned ICU admission

Variable	High Quality +'ve Association (ref)	Moderate Quality +'ve Association (ref)	Low Quality +'ve Association (ref)	Negative association (ref)	Overall	Category
History of congestive heart failure (cardiovascular disorder)	[21, 29]				Strong	Comorbidities
History of diabetes (metabolic disorder)	[21, 29]				Strong	Comorbidities
Male	[20, 21, 28]				Strong	Demographic
Increasing age	[18, 20, 21, 28]	[16]	[27]		Strong	Demographic
Diagnosis of hepatic disease (gastrointestinal disorder)	[28, 30]				Strong	Diagnosis
Higher heart rate (> 111 bpm or higher mean in ICU group)	[18, 20, 29, 30]	[16]	[27]		Strong	Vital signs
Higher respiratory rate (> 20 bpm or higher in ICU group)	[18, 20, 30]	[16]	[27]		Strong	Vital signs
Higher temperature	[18, 20]				Strong	Vital signs
Lower arterial oxygen saturation (< 94% or lower in ICU group)	[18, 20]	[16]			Strong	Vital signs
Lower diastolic blood pressure	[18, 20]	[16]	[27]		Strong	Vital signs
Lower systolic blood pressure	[18, 20]				Strong	Vital signs
Female				[18, 28]	Negative	Demographic
History of respiratory disorder	[21]		[27]		Moderate	Comorbidities
Urea (higher in ICU arm)	[20]	[24]	[27]		Moderate	Laboratory tests
White cell count (higher in ICU arm)	[20]	[24]			Moderate	Laboratory tests

The number in the boxes are references. Both studies from Tsai et al. [29, 30] come from the same patient data base. In accordance with the modified Grading System for Strength of Evidence, these two studies were only counted once (and weighted as a single high-quality study when shown together)

### Variables associated with unplanned ICU admission

Variables with a strong, moderate and negative weight of evidence for association with unplanned ICU admission are summarised in Table 3.

#### Comorbidities, demographics and diagnosis

A history of congestive heart failure and diabetes were the only comorbidities in this group. These had a significant result in two high-quality studies [21, 29] of the demographics, being male [20, 21, 27, 28] and an increasing age [16, 18, 20, 21, 27, 28] had a strong weight of evidence for association. Four studies showed a significant difference in mean or median age between ICU and control groups (higher in the ICU group) [18, 20, 21, 27] and three studies showed a significant OR or IRR for increased age quantiles with the oldest quantile being 75+ years of age [21, 28]. Hepatic disease was the only diagnosis strongly associated with unplanned ICU admission [28, 30].

#### Vital signs

All six vital signs had a strong association. Heart rate was studied 12 times across seven studies, 10 times as quantiles (seven times for tachycardia and three times for bradycardia) and twice as a comparison of means [16, 18, 20, 22, 27, 29, 30]. Seven of the tachycardia and one of the bradycardia quantiles (< 60 beats per minute) showed a positive association**.** Two high-quality studies also found a significant difference in mean heart rate (higher in the ICU group) [18, 20]. Elevated respiratory rate was evaluated eight times across six studies and had a strong weight of evidence [16, 20, 22, 27, 30]. Five of the six quantiles showed a significant result [16, 27, 30] and both high-quality studies examining mean respiratory rate showed a significant difference [18, 20]. The only non-significant result was a respiratory rate of > 20 breaths per minute in a low-quality study [22]. Systolic blood pressure (SBP) was evaluated seven times across five studies [16, 18, 20, 22, 27]. Two high-quality studies showed a significant reduction in mean blood pressure [18, 20] and one moderate-quality study showed a significant OR for a SBP of 80-89 mmHg versus 90 mmHg and above [16]. Diastolic blood pressure (DBP) and temperature were evaluated in the same two high-quality studies, both showing significant differences in mean (decreased for SBP and increased for temperature) [18, 20]. Both studies had very small variations, < 0.2^o^Cand < 2 mmHg respectively. Arterial oxygen saturation was studied six times across four studies [16, 18, 20, 22]. Lower saturation quantiles (< 80%, 80–89% and 90–94%) and lower mean/median saturations were shown to be significant.

Variables moderately and weakly associated with unplanned ICU admission are summarised in Table 3 and Additional file 1 (SDC-8) respectively.

## Discussion

### Statement of findings

In this systematic review of 16 observational and cohort studies evaluating ED and ward patients, we found two comorbidities (congestive cardiac failure and diabetes), two demographics (increasing age and being male), one diagnosis (hepatic disease) and six vital signs (respiratory rate, heart rate, temperature, systolic and diastolic blood pressure and arterial oxygen saturations) with a strong univariate association with unplanned ICU admission. These findings support the consensus that abnormal vital signs have significant value when predicting unplanned ICU admission. The strength of association for a history of congestive cardiac failure and diabetes and a new diagnosis of hepatic disease may reflect the high burden of care required in this patient cohort up until the terminal phase of disease. Being older and male as a risk factor for ICU admission may reflect the general hospital population as a whole. Overall this review provides a thorough summary of the candidate variables available in EPRs (and elsewhere in the clinical record) that will assist researchers to develop and evaluate predictive models for patients at risk of unplanned ICU admission.

### Clinical and research implications

Progressing from vital sign based, EWS systems to EPR based, risk model systems has incrementally improved performance, both in terms of correctly identified deteriorating ward patients (sensitivity) and the number of ‘false alarms’ generated for clinical staff (specificity and positive predictive value). These performance gains have been achieved via multivariate regression models and more recently machine learning processes [1, 32–36]. Regardless of the statistical approach, candidate variables should be selected in a methodologically robust way. In the published literature, univariate filter methods, that rank the strength of the statistical association, are among the most common [7, 8]. It is a popular approach because the univariate analysis provides a summary of the variables most likely to enhance model performance, does not involve significant computation, is relatively simple, not time consuming and produces an easily interpretable output. It does have weaknesses however, including the potential to miss variables that have no association with the outcome when evaluated *in isolation* but have an association when evaluated together with another variable (e.g. age).

Despite their performance advantage, as yet no EPR based hospital model has achieved widespread adoption. In contrast, 75% of UK National Health Service hospitals monitor ward patients using the National Early Warning Score (NEWS) [37, 38]. The success of NEWS, which is a simple aggregate score that uses the univariate associations of abnormal vital signs with adverse patient outcomes, highlights the importance of interpretability and generalisability in this research and clinical domain. Advanced scoring systems that rely on complex computational processes may be difficult to interpret (and trust) for clinical staff and therefore less likely to be adopted into general use. We hope the univariate associations described will provide a convenient and intuitive reference for clinicians and researchers alike to overcome such barriers to implementation.

### Strengths and limitations

The association of the variables does not infer causality. The search strategy was thorough and in accordance with current methodological guidelines but studies may have been missed. Publication bias may have affected results. The methodology of the included studies was varied, making meta-analysis inappropriate. We excluded studies examining specific sub-populations of patients only (i.e. acute liver failure) meaning the variables summarised in this review are not applicable for risk models designed for specific disease sub-groups.

There is a lack of consensus on which outcome measures to use when assessing the performance of predictive models for clinical deterioration [18]. Each of cardiac arrest, in hospital death and unplanned ICU admissions represent different populations and will, therefore, have different variable associations. We selected unplanned ICU admission as an isolated outcome measure (and excluded in hospital death and cardiac arrest) in the knowledge this would reduce the number of eligible studies and therefore potential variables for inclusion in this review. We adopted this method because we aim to advance the study of models that predict clinical deterioration, specifically in those who will most benefit from an intervention such as an ICU admission. When predicting ICU admission, some authors published the univariate relationships from within their derivation databases before including them in the multivariate analysis [20]. However we are not aware of any who have based selection on associations evaluated in external databases.

We evaluated a heterogeneous study population by including ED, ward, post-Medical Emergency Team (MET) and non-post-MET patients. This was done because we wish to better understand associations with clinical deterioration, which may occur at any time-point during hospital admission. Namely, early deterioration, which may occur soon after discharge from ED to the ward, in which case patient centred ED data is important. Or late deterioration, when the patient has been on the ward for some days. Studies examining sub-populations of patients (i.e. where specifically designed predictive algorithms have the potential to be more accurate than when used in a general patient population) were excluded on the basis that as a first step, we wish to isolate variables that will contribute to a hospital wide EPR based risk score.

We have deliberately avoided describing multivariate studies because we do not wish describe the models themselves. There are multiple examples of high performing, multivariate clinical predictive models in the literature, whose variables will have quantifiable associations with unplanned ICU admission. However, it is impossible to exclude collinearity in these instances, making obsolete our objective to individually quantify these variable associations as potential “building blocks” for future models. As a consequence, multivariate analysis was excluded unless the univariate associations were described.

## Conclusion

Having abnormal vital signs, being elderly, male, having a history of heart failure or diabetes and a diagnosis of liver failure are all strongly associated with unplanned ICU admission. This systematic review is the first to comprehensively collate the evidence on patient centred variables with univariate associations with ICU admission. These results may assist the development of predictive models for hospitalised patients at risk of needing escalations in care. There is a lack of high-quality data in this field and further work is required to isolate the patient centred variables most likely to enhance model performance when predicting unplanned ICU admission.

## Additional file

Additional file 1: Supplementary Digital Content. SDC-1 Systematic Review Search Design. SDC-2 Studies excluded because of a single or grouped diagnosis. SDC-3 Modified Newcastle-Ottawa Scale for assessment of study quality (adapted). SDC-4 Details (additional) of included studies. SDC-5 Bias scores. SDC-6 Patient populations of included studies. SDC-7 Patient derived variables examined for an association with unplanned ICU admission. In categories and then alphabetical order. SDC-8 Strength of evidence for individual variables (weak and inconclusive results) (DOCX 90 kb)

## Abbreviations

CI: Chief Investigator
CTRG: Clinical Trials & Research Governance, University of Oxford
ED: Emergency Department
EPR: Electronic Patient Record
GCP: Good Clinical Practice
ICU: Intensive Care Unit
IRR: Incidence Rate Ratios
NEWS: National Early Warning Score
OR: Odds Ratio
REC: Research Ethics Committee
RR: Risk Ratio
RRS: Rapid Response System

## References

[1] Kipnis P, Turk BJJ, Wulf DAA (2016). Development and validation of an electronic medical record-based alert score for detection of inpatient deterioration outside the ICU. J Biomed Inform.

[2] Bailey TCC, Chen Y, Mao Y (2013). A trial of a real-time alert for clinical deterioration in patients hospitalized on general medical wards. J Hosp Med.

[3] Kang M, Churpek MM, Zadravecz FJ, Twu NM, Adhikari R, Edelson DP. Real-time risk prediction on the wards: a feasibility study. C23 Sepsis Risk Recognit Resusc. 2015;(9):A3988–8. 10.1164/ajrccm-conference.2015.191.1_MeetingAbstracts.A3988.

[4] Alvarez CA, Clark CA, Zhang S (2013). Predicting out of intensive care unit cardiopulmonary arrest or death using electronic medical record data. BMC Med Inform Decis Mak.

[5] Steyerberg E, Moons KGM, van der Windt D (2013). Prognosis research strategy (PROGRESS) series 3: prognostic model research. PLoS Med.

[6] Sanchez-Pinto LN, Venable LR, Fahrenbach J, Churpek MM (2018). Comparison of variable selection methods for clinical predictive modeling. Int J Med Inform.

[7] Walter S, Tiemeier H (2009). Variable selection: current practice in epidemiological studies. Eur J Epidemiol.

[8] Bagherzadeh-Khiabani F, Ramezankhani A, Azizi F, Hadaegh F, Steyerberg EW, Khalili D (2016). A tutorial on variable selection for clinical prediction models: feature selection methods in data mining could improve the results. J Clin Epidemiol.

[9] Malycha J, Bonnici T, Sebekova K, Petrinic T, Young D, Watkinson P (2017). Variables associated with unplanned general adult ICU admission in hospitalised patients: protocol for a systematic review. Syst Rev.

[10] Moher D, Liberati A, Tetzlaff J, et al. Preferred reporting items for systematic reviews and meta-analyses: the PRISMA statement. PLoS Med. 2009;6(7). 10.1371/journal.pmed.1000097. 10.1371/journal.pmed.1000097 PMC2707599 19621072

[11] Taggart DP, D’Amico R, Altman DG (2001). Effect of arterial revascularisation on survival: a systematic review of studies comparing bilateral and single internal mammary arteries. Lancet..

[12] Duckitt K, Harrington D (2005). Risk factors for pre-eclampsia at antenatal booking: systematic review of controlled studies. Br Med J.

[13] Wells GA, Shea B, O’Connell D, Peterson J, Welch V, Losos M et al. TN-OS (NOS) for assessing the quality of nonrandomised studies in meta-analyses. Crick Cent 2015:2018. [cited 25 Apr 2016]. A from: http://www.ohri.ca/programs/clinical_epidemiology/oxford.asp. Our Research.

[14] Dettmer MR, Damuth E, Zarbiv S, Mitchell JA, Bartock JL, Trzeciak S (2017). Prognostic factors for long-term mortality in critically ill patients treated with prolonged mechanical ventilation: a systematic review. Crit Care Med.

[15] Zaal IJ, Devlin JW, Peelen LM, Slooter AJC (2015). A systematic review of risk factors for delirium in the ICU*. Crit Care Med.

[16] Barfod C, Lauritzen MMP, Danker JK (2012). Abnormal vital signs are strong predictors for intensive care unit admission and in-hospital mortality in adults triaged in the emergency department - a prospective cohort study. Scand J Trauma Resusc Emerg Med.

[17] Calzavacca P, Licari E, Tee A, Bellomo R (2012). Point-of-care testing during medical emergency team activations: a pilot study. Resuscitation..

[18] Churpek MM, Yuen TC, Edelson DP (2013). Predicting clinical deterioration in the hospital: the impact of outcome selection. Resuscitation..

[19] Eick C, Rizas KD, Meyer-Zurn CS (2015). Autonomic nervous system activity as risk predictor in the medical emergency department: a prospective cohort study. Crit Care Med.

[20] Escobar GJ, LaGuardia JC, Turk BJ, Ragins A, Kipnis P, Draper D (2012). Early detection of impending physiologic deterioration among patients who are not in intensive care: development of predictive models using data from an automated electronic medical record. J Hosp Med.

[21] Frost SA, Alexandrou E, Bogdanovski T, Salamonson Y, Parr MJ, Hillman KM (2009). Unplanned admission to intensive care after emergency hospitalisation: risk factors and development of a nomogram for individualising risk. Resuscitation..

[22] Hong W, Earnest A, Sultana P, Koh Z, Shahidah N, Ong MEH (2013). How accurate are vital signs in predicting clinical outcomes in critically ill emergency department patients. Eur J Emerg Med.

[23] Hunziker S, Stevens J, Howell MD (2012). Red cell distribution width and mortality in newly hospitalized patients. Am J Med.

[24] Loekito E, Bailey J, Bellomo R (2013). Common laboratory tests predict imminent death in ward patients. Resuscitation..

[25] Schuetz P, Hausfater P, Amin D (2015). Biomarkers from distinct biological pathways improve early risk stratification in medical emergency patients: the multinational, prospective, observational TRIAGE study. Crit Care.

[26] Steiner D, Renetseder F, Kutz A (2016). Performance of the Manchester triage system in adult medical emergency patients: a prospective cohort study. J Emerg Med.

[27] Sudarshan M, Feldman LS, St. Louis E (2015). Predictors of mortality and morbidity for acute care surgery patients. J Surg Res.

[28] Tam V, Frost SAA, Hillman KMM, Salamonson Y (2008). Using administrative data to develop a nomogram for individualising risk of unplanned admission to intensive care. Resuscitation..

[29] Tsai JCH, Cheng C-W, Weng S-J, Huang C-Y, Yen DH-T, Chen H-L (2014). Comparison of risks factors for unplanned ICU transfer after ED admission in patients with infections and those without infections. ScientificWorldJournal..

[30] Tsai JCH, Weng SJ, Huang CY, Yen DHT, Chen HL (2014). Feasibility of using the predisposition, insult/infection, physiological response, and organ dysfunction concept of sepsis to predict the risk of deterioration and unplanned intensive care unit transfer after emergency department admission. J Chinese Med Assoc.

[31] Wunderink RG, Diederich ER, Caramez MP (2012). Rapid response team-triggered procalcitonin measurement predicts infectious intensive care unit transfers. Crit Care Med.

[32] Churpek MM, Yuen TC, Park SY, Meltzer DO, Hall JB, Edelson DP (2012). Derivation of a cardiac arrest prediction model using ward vital signs. Crit Care Med.

[33] Loekito E, Bailey J, Bellomo R (2013). Common laboratory tests predict imminent medical emergency team calls, intensive care unit admission or death in emergency department patients. Emerg Med Australas.

[34] Stelfox HT, Bagshaw SM, Gao S (2015). A retrospective cohort study of age-based differences in the care of hospitalized patients with sudden clinical deterioration. J Crit Care.

[35] Hackmann G, Chen M, Chipara O (2011). Toward a two-tier clinical warning system for hospitalized patients. AMIA Annu Symp proceedings AMIA Symp.

[36] Churpek M, Yuen T, Park SED (2015). Using electronic health record data to develop and validate a prediction model for adverse outcomes on the wards. Crit Care Med.

[37] Royal College of Physicians (2012). National Early Warning Score (NEWS). Standardising the assessment of acute-illness severity in the NHS. Report of a working party.

[38] Royal College of Physcians (2017). National Early Warning Score (NEWS) 2: Standardising the assessment of acute-illness severity in the NHS. Updated report of a working party.

