# Supplementary material for: Patient centred variables with univariate associations with unplanned ICU admission: a systematic review
Source: BMC Med Inform Decis Mak. 2019 May 15;19:98. doi: 10.1186/s12911-019-0820-1 (PMC6521409; doi:10.1186/s12911-019-0820-1)
Supplement: Supplementary file 1 — Supplementary Digital Content. SDC-1 Systematic Review Search Design. SDC-2 Studies excluded because of a single or grouped diagnosis. SDC-3 Modified Newcastle-Ottawa Scale for assessment of study quality (adapted). SDC-4 Details (additional) of included studies. SDC-5 Bias scores. SDC-6 Patient populations of included studies. SDC-7 Patient derived variables examined for an association with unplanned ICU admission. In categories and then alphabetical order. SDC-8 Strength of evidence for individual variables (weak and inconclusive results) (DOCX 90 kb) [file 12911_2019_820_MOESM1_ESM.docx]

**Additional File 1**

**SDC-1. Systematic Review Search Design.**

#1. (ICU* or "intensive care" or "critical care").ab,ti.

#2. INTENSIVE CARE UNITS/

#3. CRITICAL CARE/

#4. 1 or 2 or 3

#5. (admission* or admitted or transfer*).ab,ti.

#6. ("risk assessment*" or "risk factor*" or "risk stratif*" or

predict* or "increased risk*" or trigger* or score* or

scoring or "early warning" or escalat* or deteriorat*

or triag* or "vital sign*" or model* or validat*).ab,ti.

#7. 4 and 5 and 6

#8. limit 7 to (humans and yr="2000 Current")

#9. (observational or "case control*" or retrospective or

cohort* or "systematic review*").ab,ti.

#10. OBSERVATIONAL STUDY/

#11. CASECONTROL STUDIES/

#12. RETROSPECTIVE STUDIES/

#13. COHORT STUDIES/

#14. RANDOMIZED CONTROLLED TRIAL/

#15. REVIEW/

#16. COMPARATIVE STUDY/

#17. PROSPECTIVE STUDIES/

#18. VALIDATION STUDIES

#19. 9 or 10 or 11 or 12 or 13 or 14 or 15 or 16 or 17 or 18

#20. 8 and 19

#21. limit 20 to (article or "review")

#22. (unplanned or unexpected or unanticipated or

emergency or "rapid response").ti,ab.

#23. 21 and 22

**SDC-2. Studies excluded because of a single or grouped diagnosis.**

| A. M. Moller,T. Pedersen,N. Villebro,A. Schnaberich,M. Haas,R. Tonnesen. A study of the impact of long-term tobacco smoking on postoperative intensive care admission. Anaesthesia. 2003. 58:55-9 |
| --- |
| G. Z. Gabayan,M. K. Gould,R. E. Weiss,N. Patel,K. A. Donkor,V. Y. Chiu,S. C. Yiu,J. P. Jones,J. R. Hoffman,C. A. Sarkisian. Poor Outcomes after Emergency Department Discharge of the Elderly: A Case-Control Study. Annals of Emergency Medicine. 2016. 68:43-51 |
| J. C. Knott,S. L. Tan,A. C. Street,M. Bailey,P. Cameron. Febrile adults presenting to the emergency department: Outcomes and markers of serious illness. Emergency Medicine Journal. 2004. 21:170-174 |
| J. Deibener-Kaminsky,J. F. Lesesve,S. Grosset,L. Pruna,M. C. Schmall-Laurain,A. Benetos,P. Kaminsky. [Clinical relevance of leukocyte differential in patients with marked leukocytosis in the emergency room]. Signification d'une hyperleucocytose marquee et de la formule sanguine dans les situations d'urgence.. 2011. 32:406-10 |
| J. M. Caterino,R. A. Murden,K. B. Stevenson. Functional Status Does Not Predict Complicated Clinical Course in Older Adults in the Emergency Department with Infection. Journal of the American Geriatrics Society. 2012. 60:304-309 6p |
| L. Durairaj,B. Reilly,K. Das,C. Smith,C. Acob,S. Husain,M. Saquib,P. Ganschow,A. Evans,R. McNutt. Emergency department admissions to inpatient cardiac telemetry beds: A prospective cohort study of risk stratification and outcomes. American Journal of Medicine. 2001. 110:7-11 |
| M. Garrouste-Orgeas,A. Boumendil,D. Pateron,P. Aergerter,D. Somme,T. Simon,B. Guidet. Selection of intensive care unit admission criteria for patients aged 80 years and over and compliance of emergency and intensive care unit physicians with the selected criteria: An observational, multicenter, prospective study. Critical Care Medicine. 2009. 37:2919-2928 10p |
| M. K. Jessen,J. Mackenhauer,A. M. S. W. Hvass,U. Heide-Jorgensen,C. F. Christiansen,H. Kirkegaard. Predictors of intensive care unit transfer or death in emergency department patients with suspected infection. European Journal of Emergency Medicine. 2015. 22:176-180 |
| M. Kennedy,N. Joyce,M. D. Howell,J. L. Mottley,N. I. Shapiro. Identifying Infected Emergency Department Patients Admitted to the Hospital Ward at Risk of Clinical Deterioration and Intensive Care Unit Transfer. Academic Emergency Medicine. 2010. 17:1080-1085 6p |
| N. Peschanski,C. Chenevier-Gobeaux,L. Mzabi,R. Lucas,S. Ouahabi,V. Aquilina,V. Brunel,G. Lefevre,P. Ray. Prognostic value of PCT in septic emergency patients. Annals of Intensive Care. 2016. |
| S. Küpper,C. J. Karvellas,R. G. Khadaroo,S. L. Widder. Increased health services use by severely obese patients undergoing emergency surgery: a retrospective cohort study. Canadian Journal of Surgery. 2015. 58:41-47 7p |
| T. Olsson,L. Lind. Comparison of the rapid emergency medicine score and APACHE II in nonsurgical emergency department patients. Academic Emergency Medicine. 2003. 10:1040-1048 |
| T. Smith,D. Den Hartog,T. Moerman,P. Patka,E. M. M. Van Lieshout,N. W. L. Schep. Accuracy of an expanded early warning score for patients in general and trauma surgery wards. British Journal of Surgery. 2012. 99:192-197 |

**SDC-3.** **Modified** **Newcastle-Ottawa Scale for assessment of study quality (adapted).**

| **Participant Selection** | **Score** |
| --- | --- |
| **Cohort studies** |  |
| Selected cohort is *very representative* of the general hospitalised population | **2** |
| *(Ward patients scored 2 points)* |  |
| Selected cohort is *somewhat representative* of the general hospitalised population | **1** |
| *(ED patients, ward patients recruited via RRS and ward patients with a surgical diagnosis scored 1 point)* |  |
| Cohort is not representative of the general hospital population or the selection of the group was not described | **0** |
| *(No such papers are included in this review because they were excluded as per our criteria)* |  |
| **Case-control studies** |  |
| Cases and controls drawn from the same population and population is *very representative* of the general hospitalised population | **2** |
| *(Ward patients scored 2 points)* |  |
| Cases and controls drawn from the same population and population is *somewhat representative* of the general hospitalised population | **1** |
| *(ED patients and ward patients recruited via RRS scored 1 point)* |  |
| Cases and controls drawn from different sources or the selection of groups was not described | **0** |
| *(No such papers are included in this review because they were excluded as per our criteria)* |  |
| **Comparability of groups** |  |
| No differences between the groups explicitly reported unless it was one of the variables that was under investigation, or such differences were adjusted for | **2** |
| *(The ICU and control arms had to be compared directly and show not to be different to score 2 points)* |  |
| Differences between groups were not recorded | **1** |
| *(If the ICU and control arms were not described, the assumption being they may or may not have differed)* |  |
| Groups differed | **0** |
| *(If the ICU and control arms were described and shown to be different)* |  |
| **Size** |  |
| > 100 participants in each group | **2** |
| < 100 participants in each group | **1** |
| **Confounding** |  |
| Adjusted for confounders in study methodology | **2** |
| *(Patient selection and recruitment was non biased)* |  |
| Adjusted for confounders during data analysis | **1** |
| *(Once selected the ICU and control arm variables underwent univariate analysis with any adjustment described in the methodology)* |  |
| Did not adjust for confounders | **0** |
| *(No methodological or analytical description of adjustment)* |  |

**SDC-4. Details (additional) of included studies**

| **Ref** | **Lead Author** | **Study type** | **Prospective or Retrospective** | **ICU admission primary or secondary endpoint?** | **Method of variable selection** | **Cohort** |
| --- | --- | --- | --- | --- | --- | --- |
| 16 | Barfod | Cohort | Prospective | Primary | Based on existing scores | Ward patients admitted via ED |
| 17 | Calzavacca | Cohort | Prospective | Secondary | Defined by testing device | Ward patients reviewed by RRS team |
| 18 | Churpek | Cohort | Retrospective | Primary | Not specified | Ward patients |
| 19 | Eick | Cohort | Prospective | Secondary | Scientific hypothesis | ED patients |
| 20 | Escobar | Case control | Retrospective | Primary | Derived from previous study by same author | Ward patients |
| 21 | Frost | Case control | Retrospective | Primary | Based on existing scores | Ward patients |
| 22 | Hong | Cohort | Prospective | Primary | Dervided from Patient Acuity Category traige scale | ED patients |
| 23 | Hunziker | Case control | Prospective | Secondary | Scientific hypothesis | Ward patients |
| 24 | Loekiko | Cohort | Retrospective | Primary | Statistically selected from a list of 30 lab tests as most predictive of death | ED patients |
| 25 | Schuetz | Cohort | Prospective | Secondary | Scientific hypothesis | ED patients |
| 26 | Steiner | Cohort | Prospective | Secondary | Based on existing scores | ED patients |
| 27 | Sudarshan | Cohort | Retrospective | Primary | Not specified | Emergency surgical admissions ward patients |
| 28 | Tam | Cohort | Retrospective | Primary | Not specified | Ward patients |
| 29 | Tsai | Case control | Retrospective | Primary | Based on existing scores | Ward patients admitted via ED |
| 30 | Tsai | Case control | Retrospective | Primary | Based on existing scores | Ward patients admitted via ED |
| 31 | Wunderink | Cohort | Prospective | Primary | Scientific hypothesis | Ward patients reviewed by RRS team |

**SDC-5. Bias scores**

| Reference | Total | Score of cohort represent-ativeness | Score for group differences | Score for group sizes | Score for adjustment for confounding |
| --- | --- | --- | --- | --- | --- |
| 16 | **5 - MQ** | 1 | 2 | 2 | 0 |
| 17 | **2 - LQ** | 1 | 1 | 0 | 0 |
| 18 | **7 - HQ** | 2 | 2 | 2 | 1 |
| 19 | **4 - LQ** | 1 | 1 | 2 | 0 |
| 20 | **7 - HQ** | 2 | 2 | 2 | 1 |
| 21 | **7 - HQ** | 2 | 2 | 2 | 1 |
| 22 | **4 - LQ** | 1 | 1 | 2 | 0 |
| 23 | **5 - MQ** | 2 | 1 | 2 | 0 |
| 24 | **5 - MQ** | 1 | 1 | 2 | 1 |
| 25 | **5 - MQ** | 1 | 1 | 2 | 1 |
| 26 | **4 - LQ** | 1 | 1 | 2 | 0 |
| 27 | **3 - LQ** | 1 | 1 | 1 | 0 |
| 28 | **7 - HQ** | 2 | 2 | 2 | 1 |
| 29 | **7 - HQ** | 2 | 2 | 2 | 1 |
| 30 | **7 - HQ** | 2 | 2 | 2 | 1 |
| 31 | **3 - LQ** | 1 | 1 | 1 | 0 |

**SDC-6. Patient populations of included studies**

| **Ref** | **ED patient** | **Ward patient admitted via ED** | **Ward patient admitted from any source** | **Ward patient reviewed by RRT/MET** |
| --- | --- | --- | --- | --- |
| 16 |  | * |  |  |
| 17 |  |  |  | * |
| 18 |  |  | * |  |
| 19 | * |  |  |  |
| 20 |  |  | * |  |
| 21 |  | * |  |  |
| 22 | * |  |  |  |
| 23 |  |  | * |  |
| 24 | * |  |  |  |
| 25 | * |  |  |  |
| 26 | * |  |  |  |
| 27 |  | * |  |  |
| 28 |  |  | * |  |
| 29 |  | * |  |  |
| 30 |  | * |  |  |
| 31 |  |  |  | * |

ED: Emergency Departmant

RRT: Rapid Response Team

MET: Medical Emergency Team

**SDC-7. Patient derived variables examined for an association with unplanned ICU admission. In categories and then alphabetical order.**

| **Ref** | **Study quality** | **Variable** | **Variable type** | **ICU vs Control p value** | **OR** | **95%CI** |
| --- | --- | --- | --- | --- | --- | --- |
| 28 | LQ | Alcohol consumption (history of) | Comorbidities | Non significant |  |  |
| 28 | LQ | ASA Class > 3 [lower in ICU arm] | Comorbidities | <0.001 |  |  |
| 21 | HQ | Cardiovascular disorder, acute MI (history of) | Comorbidities | 0.2 | 1.2 | 1.0–1.4 |
| 28 | LQ | Cardiovascular disorder, arrhythmia (history of) | Comorbidities | Non significant |  |  |
| 21 | HQ | Cardiovascular disorder, congestive heart failure (history of) | Comorbidities | 0.02 | 1.5 | 1.3–1.7 |
| 30 | HQ | Cardiovascular disorder, congestive heart failure (history of) | Comorbidities | 0.001 | 2.2 | 1.4–3.4 |
| 28 | LQ | Cardiovascular disorder, coronary artery disease (history of) | Comorbidities | Non significant |  |  |
| 30 | HQ | Cardiovascular disorder, coronary artery disease (history of) | Comorbidities | 0.02 | 1.7 | 1.1–2.5 |
| 28 | LQ | Cardiovascular disorder, hypertension (history of) | Comorbidities | Non significant |  |  |
| 21 | HQ | Cardiovascular disorder, peripheral vascular disease (history of) | Comorbidities | <0.001 | 1.6 | 1.3–1.9 |
| 21 | *HQ* | *Connective tissue disorder (history of)* | *Comorbidities* | *<0.001* | *1.4* | *1.0–2.0* |
| 21 | HQ | Gastrointestinal disorder, hepatic disease (severe) (history of) | Comorbidities | 0.2 | 1.3 | 0.8–2.0 |
| 30 | HQ | Gastrointestinal disorder, hepatic disease (severe) (history of) | Comorbidities | <0.001 | 4.4 | 2.5–7.9 |
| 21 | *HQ* | *Gastrointestinal disorder, hepatic disease [history of)* | *Comorbidities* | *1* | *1.4* | *1.1–1.9* |
| 30 | HQ | Gastrointestinal disorder, hepatic dysfunction (history of) | Comorbidities | 0.11 | 2.6 | 0.8–7.7 |
| 28 | LQ | Gastrointestinal disorder, hepatobiliary disease (history of) | Comorbidities | Non significant |  |  |
| 21 | HQ | Gastrointestinal disorder, peptic ulcer disease (history of) | Comorbidities | 0.05 | 1 | 0.8–1.3 |
| 21 | HQ | Infective disorder, HIV (history of) | Comorbidities | NA |  |  |
| 30 | HQ | Infective disorder, immunocompromise (history of) | Comorbidities | 0.19 | 2.1 | 0.7–6.8 |
| 28 | LQ | Malignancy (history of) | Comorbidities | Non significant |  |  |
| 21 | HQ | Malignancy (history of) | Comorbidities | <0.001 | 1.4 | 1.2–1.7 |
| 30 | HQ | Malignancy, advanced (history of) | Comorbidities | 0.54 | 1.2 | 0.7–2.2 |
| 21 | *HQ* | *Malignancy, metastatic (history of)* | *Comorbidities* | *<0.001* | *1.2* | *0.9–1.6* |
| 21 | HQ | Metabolic disorder, diabetes (history of) | Comorbidities | 0.001 | 1.3 | 1.2–1.5 |
| 30 | HQ | Metabolic disorder, diabetes (history of) | Comorbidities | 0.002 | 1.8 | 1.3–2.6 |
| 28 | LQ | Metabolic disorder, diabetes type 2 (history of) | Comorbidities | Non significant |  |  |
| 21 | HQ | Metabolic disorder, diabetes with complications (history of) | Comorbidities | <0.001 | 1.3 | 1.1–1.6 |
| 28 | LQ | Metabolic disorder, endocrine disease (history of) | Comorbidities | Non significant |  |  |
| 30 | HQ | Neurological disorder, cerebral performance category of 3 or 4 (history of) | Comorbidities | <0.001 | 3.3 | 1.9–5.7 |
| 28 | LQ | Neurological disorder, cerebrovascular disease (history of) | Comorbidities | Non significant |  |  |
| 30 | HQ | Neurological disorder, cerebrovascular disease (history of) | Comorbidities | 0.08 | 1.5 | 1.0–2.3 |
| 21 | HQ | Neurological disorder, cerebrovascular disease (history of) | Comorbidities | <0.001 | 1.3 | 1.1–1.5 |
| 28 | LQ | Neurological disorder, dementia (history of) | Comorbidities | 0.01 |  |  |
| 21 | HQ | Neurological disorder, dementia (history of) | Comorbidities | 0.01 | 0.8 | 0.7–0.9 |
| 21 | *HQ* | *Neurological disorder, paraplegia (history of)* | *Comorbidities* | *0.006* | *1.1* | *0.9–1.4* |
| 21 | HQ | *Renal disorder (history of)* | *Comorbidities* | *0.2* | *1.7* | *1.4–2.0* |
| 28 | LQ | Renal disorder, chronic kidney disease (history of) | Comorbidities | Non significant |  |  |
| 30 | HQ | Renal disorder, end-stage renal disease (history of) | Comorbidities | 0.03 | 2.5 | 1.2–5.3 |
| 21 | HQ | Respiratory disorder (history of) | Comorbidities | 0.007 | 1.4 | 1.2–1.6 |
| 28 | LQ | Respiratory disorder (history of) | Comorbidities | 0.02 |  |  |
| 30 | HQ | Respiratory disorder, respiratory failure (history of) | Comorbidities | <0.001 | 7.3 | 2.8–19.4 |
| 28 | LQ | Smoking status, Ex-Smoker (history of) | Comorbidities | Non significant |  |  |
| 16 | MQ | Age (yrs) [OR per year] | Demographic |  | 1.02 | 1.01–1.03 |
| 30 | HQ | Age, ≥65 (yrs) [more in this demographic in ICU group] | Demographic | 0.14 | 1.3 | 0.9–1.8 |
| 21 | HQ | Age, 25-34 (yrs) [IRR reference 15-24] | Demographic |  | 1^ | 0.1-1.3 |
| 29 | HQ | Age, 30-44 (yrs) [IRR reference 15-29] | Demographic |  | 1.7^ | 1.2-2.4 |
| 21 | HQ | Age, 35-44 (yrs) [IRR reference 15-24] | Demographic |  | 1.3^ | 0.9-1.7 |
| 21 | HQ | Age, 45-54 (yrs) [IRR reference 15-24] | Demographic |  | 2.2^ | 1.7-2.9 |
| 29 | HQ | Age, 45-59 (yrs) [IRR reference 15-29] | Demographic |  | 4.0^ | 2.9-5.6 |
| 21 | HQ | Age, 55-64 (yrs) [IRR reference 15-24] | Demographic |  | 2.9^ | 2.3-3.9 |
| 29 | HQ | Age, 60-74 (yrs) [IRR reference 15-29] | Demographic |  | 9.0^ | 6.6-12.2 |
| 21 | HQ | Age, 65-74 (yrs) [IRR reference 15-24] | Demographic |  | 3.9^ | 3.1-5.1 |
| 21 | HQ | Age, 75+ (yrs) [IRR reference 15-24] | Demographic |  | 3.2^ | 2.5-4.2 |
| 29 | HQ | Age, 75+ (yrs) [IRR reference 15-29] | Demographic |  | 10.5^ | 7.7-14.2 |
| 21 | HQ | Age, mean (yrs) [higher in ICU group] | Demographic | <0.001 |  |  |
| 18 | HQ | Age, mean (yrs)[higher in ICU group] | Demographic | <0.05 |  |  |
| 20 | HQ | Age, mean (yrs)[higher in ICU group] | Demographic | <0.001 |  |  |
| 28 | LQ | Age, median (yrs) [higher in ICU group] | Demographic | 0.0046 |  |  |
| 29 | HQ | Gender, Female (%) [lower in ICU group] | Demographic | <0.001 |  |  |
| 18 | HQ | Gender, Female (%) [lower in ICU group] | Demographic | <0.05 |  |  |
| 28 | LQ | Gender, Male (%)[higher in ICU group] | Demographic | Non significant |  |  |
| 20 | HQ | Gender, Male (%)[increased in ICU group] | Demographic | <0.001 |  |  |
| 21 | HQ | Gender, Male [IRR reference female] | Demographic | <0.001 | 1.38^ | 1.3-1.5 |
| 29 | HQ | Gender, Male [IRR reference female] | Demographic |  | 1.7^ | 1.5-2.0 |
| 18 | HQ | Race, Black (%) | Demographic | Not significant |  |  |
| 18 | HQ | Race, Other (%) | Demographic | Not significant |  |  |
| 18 | HQ | Race, Unknown (%) | Demographic | Not significant |  |  |
| 18 | HQ | Race, White (%) | Demographic | Not significant |  |  |
| 26 | LQ | Cardiovascular disorder (diagnosis of) | Diagnosis |  | 2.9 | 1.7–4.9 |
| 29 | HQ | Cardiovascular disorder, acute myocardial infarction (history of) [higher in ICU arm] | Diagnosis | <0.001 | 2.6^ | 1.8-3.8 |
| 29 | HQ | Cardiovascular disorder, congestive heart failure (history of) | Diagnosis | <0.001 | 2.8^ | 1.8-4.3 |
| 30 | HQ | Cardiovascular disorder, hypotension (diagnosis of) | Diagnosis | <0.001 | 4 | 2.0–7.9 |
| 31 | HQ | Cardiovascular disorder, hypotension (diagnosis of) | Diagnosis |  | 5 | 2.7–9.3 |
| 19 | LQ | Deceleration Capacity, mean (m/s) (lower in ICU group) | Diagnosis | <0.001 |  |  |
| 29 | HQ | Femur (fracture) [higher in ICU arm][OR reference no fracture] | Diagnosis | <0.001 | 5.7^ | 3.8-8.6 |
| 26 | LQ | Gastrointestinal disorder (diagnosis of) | Diagnosis |  | 2 | 0.4–9.5 |
| 29 | HQ | Gastrointestinal disorder, hepatic disease disease [higher in ICU arm][IRR reference no disease] (diagnosis of) | Diagnosis | <0.001 | 11.1^ | 5.9-20.9 |
| 31 | HQ | Gastrointestinal disorder, hepatic dysfunction (diagnosis of) | Diagnosis |  | 6.3 | 1.6–23.8 |
| 29 | HQ | Gastrointestinal disorder, pancreatitis (diagnosis of) [IRR reference no pancreatitis] | Diagnosis |  | 4.1^ | 2.4-7.2 |
| 30 | HQ | Haematological dysfunction (diagnosis of) | Diagnosis | <0.001 | 3.7 | 2.1–6.7 |
| 31 | HQ | Haematological dysfunction (diagnosis of) | Diagnosis |  | 4.4 | 2.4–8.1 |
| 26 | LQ | Infective disorder (diagnosis of) | Diagnosis |  | 2.2 | 1.1–4.5 |
| 26 | LQ | Malignancy (diagnosis of) | Diagnosis |  | 3.6 | 0.6–22.2 |
| 26 | LQ | Metabolic disorder (diagnosis of) | Diagnosis |  | 1.2 | 0.1–9.5 |
| 30 | HQ | Metabolic disorder, dysfunction (diagnosis of) | Diagnosis | <0.001 | 8.1 | 2.9–23.1 |
| 31 | HQ | Metabolic disorder, dysfunction (diagnosis of) | Diagnosis |  | 7.4 | 2.9–18.9 |
| 26 | LQ | Neurological disorder (diagnosis of) | Diagnosis |  | 7.8 | 3.3–18.6 |
| 29 | HQ | Neurological disorder, cerebral infarction (diagnosis of) [higher in ICU arm][IRR reference no disease] | Diagnosis | <0.001 | 3.4^ | 1.9-6.1 |
| 26 | LQ | Other Diagnosis | Diagnosis |  | 3 | 1.1–7.8 |
| 30 | HQ | Renal disorder, dysfunction (diagnosis of) | Diagnosis | 0.002 | 2.3 | 1.4–3.8 |
| 31 | HQ | Renal disorder, dysfunction (diagnosis of) | Diagnosis |  | 2.9 | 1.7–4.7 |
| 26 | LQ | Respiratory disorder (diagnosis of) | Diagnosis |  | 1.7 | 0.5–5.6 |
| 29 | HQ | Respiratory disorder, asthma (diagnosis of)[IRR reference no disease] | Diagnosis |  | 0.8^ | 0.3-2.0 |
| 29 | HQ | Respiratory disorder, COPD (diagnosis of) [higher in ICU arm][IRR reference no disease] | Diagnosis | <0.001 | 3.6^ | 2.5-4.5 |
| 30 | HQ | Respiratory disorder, dysfunction (diagnosis of) | Diagnosis | <0.001 | 4.3 | 2.3–8.0 |
| 31 | HQ | Respiratory disorder, dysfunction (diagnosis of) | Diagnosis |  | 3.3 | 2.0–5.6 |
| 29 | HQ | Respiratory disorder, pneumonia (diagnosis of) [higher in ICU arm][IRR reference no disease] | Diagnosis | <0.001 | 4.4^ | 3.3-5.8 |
| 24 | MQ | Albumin, mean (g/L) [lower in ICU arm][OR threshold ≤ 33.5] | Laboratory tests | <0.0001 | 5.32 | 3.88-7.3 |
| 24 | *MQ* | *Bilirubin, median (umol/L) [higher in ICU arm][OR threshold ≥ 14.5]* | *Laboratory tests* | *0.59* | *1.68* | *1.22-2.33* |
| 17 | LQ | BNP, mean (ng/ml) | Laboratory tests | Not reported |  |  |
| 25 | MQ | Copeptin, increase in (pmol/L) | Laboratory tests |  | 3 | 2.6–3.4 |
| 28 | LQ | Creatinine, median (mmol/L) | Laboratory tests | Non significant |  |  |
| 24 | MQ | Creatinine, median (mmol/L) [higher in ICU arm][OR threshold ≥ 105.5] | Laboratory tests | <0.0001 | 6.04 | 4.51-8.09 |
| 17 | LQ | D-dimer, mean (ng/ml) | Laboratory tests | Not reported |  |  |
| 24 | MQ | Haematocrit, mean (L/L) [lower in ICU arm][OR threshold ≤ 0.345] | Laboratory tests | 0.005 | 2.3 | 1.69-3.12 |
| 20 | HQ | Haematocrit, mean [lower in ICU arm] | Laboratory tests | <0.103 |  |  |
| 24 | MQ | Haemoglobin, mean (g/L) [lower in ICU arm][OR threshold ≥ 106.5] | Laboratory tests | <0.0001 | 3.07 | 2.22-4.24 |
| 24 | MQ | pH, mean [lower in ICU arm][OR threshold ≤ 7.315] | Laboratory tests | <0.0001 | 7.68 | 6.04-9.78 |
| 25 | MQ | Pro-adrenomedullin, increase in (nmol/L) | Laboratory tests |  | 7.7 | 5.8–10.3 |
| 25 | MQ | Procalcitonin, increase in (ug/L) | Laboratory tests |  | 1.9 | 1.6–2.1 |
| 32 | LQ | Procalcitonin, median (ng/ml) [higher in ICU arm] | Laboratory tests | <0.0001 |  |  |
| 23 | MQ | Red cell distribution width, increase in deciles (%) | Laboratory tests |  | 1.15 | 1.14–1.17 |
| 24 | MQ | Total bicarbonate, mean (mmol/L) [lower in ICU arm] | Laboratory tests | <0.0001 |  |  |
| 20 | HQ | Urea, mean (mmol/L) [higher in ICU arm] | Laboratory tests | <0.001 |  |  |
| 28 | LQ | Urea, median (mmol/L) [higher in ICU arm] | Laboratory tests | 0.0219 |  |  |
| 24 | MQ | Urea, median (mmol/L) [higher in ICU arm][OR threshold ≥ 9.05] | Laboratory tests | <0.0001 | 3.54 | 2.64-4.75 |
| 28 | LQ | White cell count, <12 (10x9/L) | Laboratory tests | Non significant |  |  |
| 28 | LQ | White cell count, <4 (10x9/L) [higher % of in ICU arm] | Laboratory tests | <0.001 |  |  |
| 30 | HQ | White cell count, >12,000 or <4000 (/uL) or band > 5 (%) (diagnosis of) | Laboratory tests | <0.001 | 3.9 | 2.1–7.3 |
| 31 | HQ | White cell count, >12,000 or <4000 (/uL) or band > 5 (%) (diagnosis of) | Laboratory tests |  | 1.7 | 1.2–2.4 |
| 20 | HQ | White cell count, mean (10x9/L) [higher in ICU arm] | Laboratory tests | <0.001 |  |  |
| 24 | *MQ* | *White cell count, median (10x9/L) [higher in ICU arm][OR threshold ≥ 12.05]* | *Laboratory tests* | *<0.47* | *3.18* | *2.38-4.24* |
| 28 | LQ | Medication, Anti-Coagulant (patient taking) | Medication | <0.001 |  |  |
| 28 | LQ | Medication, Anti-Platelet (patient taking) | Medication | Non significant |  |  |
| 28 | LQ | Medication, Corticosteriod (patient taking) | Medication | 0.076 |  |  |
| 28 | LQ | Medication, Total count of (median) [higher in ICU arm] | Medication | 0.0022 |  |  |
| 30 | HQ | Cardiovascular disorder, chest pain (new onset) (symptom/signs of) | Symptom/Sign | 0.003 | 4.4 | 1.6–11.7 |
| 26 | LQ | Infective disorder, fever (complaint of) | Symptom/Sign |  | 1.6 | 0.5–5.1 |
| 26 | LQ | Neurological disorder (symptom of) | Symptom/Sign |  | 7.5 | 3.2–17.5 |
| 30 | HQ | Neurological disorder, altered mental status (symptom/signs of) | Symptom/Sign | <0.001 | 31 | 7.1–134.6 |
| 30 | HQ | Neurological disorder, seizure (symptom/signs of) | Symptom/Sign | 0.02 | 3 | 1.2–7.4 |
| 26 | LQ | Other Presenting Complaint | Symptom/Sign |  | 6.2 | 1.7–23.0 |
| 26 | LQ | Pain, except chest pain (symptom of) | Symptom/Sign |  | 2.7 | 1.5–5.0 |
| 26 | LQ | Respiratory disorder (symptom of) | Symptom/Sign |  | 1.6 | 0.9–3.0 |
| 30 | HQ | Respiratory disorder (symptoms/signs of) | Symptom/Sign | <0.001 | 4.5 | 2.4–8.4 |
| 26 | LQ | Thoracic pain (symptom of) | Symptom/Sign |  | 3.3 | 1.4–7.8 |
| 16 | MQ | Arterial oxygen saturation, <80 (%) [OR reference 95-100] | Vital signs |  | 8.42 | 1.88–36.17 |
| 22 | LQ | Arterial oxygen saturation, <95 (%) [OR reference ≥ 95] | Vital signs |  | 1.32 | 0.88-1.97 |
| 16 | MQ | Arterial oxygen saturation, 80-89 (%) [OR reference 95-100] | Vital signs |  | 7.49 | 3.86–14.51 |
| 16 | MQ | Arterial oxygen saturation, 90-94 (%) [OR reference 95-100] | Vital signs |  | 2.35 | 1.31–4.20 |
| 20 | HQ | Arterial oxygen saturation, mean (%) [lower in ICU group] | Vital signs | <0.001 |  |  |
| 18 | HQ | Arterial oxygen saturation, median [lower in ICU group] | Vital signs | <0.05 |  |  |
| 22 | LQ | Blood pressure, diastolic, <60 (mmHg) [OR reference 60-95] | Vital signs |  | 1 | 0.67-1.49 |
| 22 | LQ | Blood pressure, diastolic, >95 (mmHg) [OR reference 60-95] | Vital signs |  | 1.13 | 0.76-1.70 |
| 18 | HQ | Blood pressure, diastolic, mean (mmHg) [lower in ICU group] | Vital signs | <0.05 |  |  |
| 20 | HQ | Blood pressure, diastolic, mean (mmHg) [lower in ICU group] | Vital signs | <0.001 |  |  |
| 16 | MQ | Blood pressure, systolic, <80 (mmHg) [OR reference 90-] | Vital signs |  | 3.22 | 0.43–24.32 |
| 28 | LQ | Blood pressure, systolic, <90 (mmHg) [lower in ICU group] | Vital signs | 0.002 |  |  |
| 22 | LQ | Blood pressure, systolic, <90 (mmHg) [OR reference 90-140] | Vital signs |  | 0.95 | 0.57-1.56 |
| 22 | LQ | Blood pressure, systolic, >140 (mmHg) [OR reference 90-140] | Vital signs |  | 0.74 | 0.53-1.03 |
| 16 | MQ | Blood pressure, systolic, 80-89 (mmHg) [OR reference 90-] | Vital signs |  | 4.97 | 1.50–16.38 |
| 18 | HQ | Blood pressure, systolic, mean (mmHg) [lower in ICU group] | Vital signs | <0.05 |  |  |
| 20 | HQ | Blood pressure, systolic, mean (mmHg) [lower in ICU group] | Vital signs | <0.001 |  |  |
| 22 | LQ | Glasgow Coma Scale, <15 (out of 15) [OR reference 15/15] | Vital signs |  | 2.41 | 1.63-3.55 |
| 16 | MQ | Glasgow Coma Scale, <8 (out of 15) [OR reference 15/15] | Vital signs |  | 5.21 | 5.24–24.91 |
| 16 | MQ | Glasgow Coma Scale, 14 (out of 15) [OR reference 15/15] | Vital signs |  | 3.57 | 1.82–7.00 |
| 16 | MQ | Glasgow Coma Scale, 9-13 (out of 15) [OR reference 15/15] | Vital signs |  | 2.02 | 0.73–5.61 |
| 16 | MQ | Heart rate, <40 (bpm) [OR reference 50-110] | Vital signs |  | 6.51 | 0.84–50.50 |
| 22 | LQ | Heart rate, <60 (bpm) [OR reference 60-100] | Vital signs |  | 2.25 | 1.32-3.81 |
| 28 | LQ | Heart rate, >100 (bpm) [lower in ICU group] | Vital signs | 0.007 |  |  |
| 22 | LQ | Heart rate, >100 (bpm) [OR reference 60-100] | Vital signs |  | 0.95 | 0.68-1.32 |
| 16 | MQ | Heart rate, >130 (bpm) [OR reference 50-110] | Vital signs |  | 8.14 | 4.07–16.28 |
| 30 | HQ | Heart rate, ≥130 (bpm) | Vital signs | 0.03 | 1.6 | 1.1–2.3 |
| 31 | HQ | Heart rate, ≥130 (bpm) | Vital signs |  | 3.7 | 2.1–6.4 |
| 16 | MQ | Heart rate, 111-120 (bpm) [OR reference 50-110] | Vital signs |  | 3.83 | 2.05–7.18 |
| 16 | MQ | Heart rate, 121-130 (bpm) [OR reference 50-110] | Vital signs |  | 8.91 | 4.68–16.95 |
| 16 | MQ | Heart rate, 40-49 (bpm) [OR reference 50-110] | Vital signs |  | 1.41 | 0.20–10.33 |
| 18 | HQ | Heart rate, mean (bpm) [higher in ICU group] | Vital signs | <0.05 |  |  |
| 20 | HQ | Heart rate, mean (bpm) [higher in ICU group] | Vital signs | <0.001 |  |  |
| 18 | HQ | Mental status, Alert [AVPU scale] | Vital signs | Non significant |  |  |
| 18 | HQ | Mental status, Responsive to pain [AVPU scale] | Vital signs | Non significant |  |  |
| 18 | HQ | Mental status, Responsive to voice [AVPU scale] | Vital signs | Non significant |  |  |
| 18 | HQ | Mental status, Unresponsive [AVPU scale] | Vital signs | Non significant |  |  |
| 28 | LQ | Respiratory rate, >20 (bpm) (% above or below 20 bpm)[lower in ICU group] | Vital signs | <0.001 |  |  |
| 22 | LQ | Respiratory rate, >20 (bpm) [OR reference 12-20] | Vital signs |  | 1.06 | 0.70-1.60 |
| 16 | MQ | Respiratory rate, >35 (bpm) [OR reference 8-25] | Vital signs |  | 9.11 | 3.49–23.80 |
| 31 | HQ | Respiratory rate, ≥30 (bpm) | Vital signs |  | 3.2 | 1.7–6.0 |
| 16 | MQ | Respiratory rate, 26-30 (bpm) [OR reference 8-25] | Vital signs |  | 3.76 | 1.97–7,20 |
| 16 | MQ | Respiratory rate, 31-35 (bpm) [OR reference 8-25] | Vital signs |  | 5.92 | 2.08–16.87 |
| 18 | HQ | Respiratory rate, mean (bpm) [higher in ICU group] | Vital signs | <0.05 |  |  |
| 20 | HQ | Respiratory rate, mean (bpm) [higher in ICU group] | Vital signs | <0.001 |  |  |
| 31 | HQ | Temperature > 38 or < 36 (degress C) | Vital signs |  | 0.8 | 0.6-1.2 |
| 28 | LQ | Temperature, <36 (degrees C) | Vital signs | Non significant |  |  |
| 28 | LQ | Temperature, >38 (degrees C) | Vital signs | Non significant |  |  |
| 18 | HQ | Temperature, mean (degrees C) [higher in ICU group] | Vital signs | <0.05 |  |  |
| 20 | HQ | Temperature, mean (degrees F) [higher in ICU group] | Vital signs | 0.009 |  |  |
| 16 | MQ | Vital sign count (1 abnormal vital sign) [OR reference 0 abnormal vital signs] | Vital signs |  | 2.2 | 1.25–3.89 |
| 16 | MQ | Vital sign count (2 abnormal vital signs) [OR reference 0 abnormal vital signs] | Vital signs |  | 13.03 | 7.64–22.23 |
| 16 | MQ | Vital sign count (3 abnormal vital signs) [OR reference 0 abnormal vital signs] | Vital signs |  | 15.99 | 6.76–37.77 |

If only one of p value and RRs/ORs/IRRs were significant, the variable was considered significant.

Black denotes a significant p value < 0.05 or Odds Ratio, Incidence Rate Ratio, Risk Ratio

Grey denotes a non-significant p value < 0.05 or Odds Ratio, Incidence Rata Ratio, Risk Ratio

*Italics denotes a difference in significance of p value < 0.05 and OR/RR/IRR*

^Denotes the value is an Incidence rate ratio, Risk Ratio [29]

**SDC-8. Strength of evidence for individual variables (weak and inconclusive results)**

| **Variable** | **High Quality +'ve Association** | **Moderate Quality +'ve Association** | **Low Quality +'ve Association** | **Negative** | **Overall** | **Category** |
| --- | --- | --- | --- | --- | --- | --- |
| Cardiovascular disorder, coronary artery disease (history of) | 29 |  |  |  | Weak | Comorbidities |
| Cardiovascular disorder, peripheral vascular disease (history of) | 21 |  |  |  | Weak | Comorbidities |
| Connective tissue disorder (history of) | 21 |  |  |  | Weak | Comorbidities |
| Gastrointestinal disorder, hepatic disease (severe) (history of) | 29 |  |  |  | Weak | Comorbidities |
| Gastrointestinal disorder, hepatic disease [history of) | 21 |  |  |  | weak | Comorbidities |
| Malignancy (history of) | 21 |  |  |  | Weak | Comorbidities |
| Malignancy, metastatic (history of) | 21 |  |  |  | Weak | Comorbidities |
| Metabolic disorder, diabetes with complications (history of) | 21 |  |  |  | Weak | Comorbidities |
| Neurological disorder, cerebral performance category of 3 or 4 (history of) | 29 |  |  |  | Weak | Comorbidities |
| Neurological disorder, cerebrovascular disease (history of) | 21 |  |  |  | Weak | Comorbidities |
| Neurological disorder, paraplegia (history of) | 21 |  |  |  | Weak | Comorbidities |
| Renal disorder (history of) | 21 |  |  |  | Weak | Comorbidities |
| Renal disorder, end-stage renal disease (history of) | 29 |  |  |  | Weak | Comorbidities |
| Respiratory disorder, respiratory failure (history of) | 29 |  |  |  | Weak | Comorbidities |
| Cardiovascular disorder, acute myocardial infarction (history of) [higher in ICU arm] | 28 |  |  |  | Weak | Diagnosis |
| Cardiovascular disorder, congestive heart failure (history of) | 28 |  |  |  | Weak | Diagnosis |
| Cardiovascular disorder, hypotension (diagnosis of) | 29, 30 |  |  |  | Weak | Diagnosis |
| Femur (fracture) [higher in ICU arm][OR reference no fracture] | 28 |  |  |  | Weak | Diagnosis |
| Gastrointestinal disorder, pancreatitis (diagnosis of) [IRR reference no pancreatitis] | 28 |  |  |  | Weak | Diagnosis |
| Haematological dysfunction (diagnosis of) | 29, 30 |  |  |  | Weak | Diagnosis |
| Metabolic disorder, dysfunction (diagnosis of) | 29, 30 |  |  |  | Weak | Diagnosis |
| Neurological disorder, cerebral infarction (diagnosis of) [higher in ICU arm][IRR reference no disease] | 28 |  |  |  | Weak | Diagnosis |
| Renal disorder, dysfunction (diagnosis of) | 29, 30 |  |  |  | Weak | Diagnosis |
| Respiratory disorder, COPD (diagnosis of) [higher in ICU arm][IRR reference no disease] | 28 |  |  |  | Weak | Diagnosis |
| Respiratory disorder, dysfunction (diagnosis of) | 29, 30 |  |  |  | Weak | Diagnosis |
| Respiratory disorder, pneumonia (diagnosis of) [higher in ICU arm][IRR reference no disease] | 28 |  |  |  | Weak | Diagnosis |
| Procalcitonin, median (ng/ml) [higher in ICU arm] |  | 25 | 31 |  | Weak | Laboratory tests |
| White cell count, >12,000 or <4000 (/uL) or band > 5 (%) | 29, 30 |  |  |  | Weak | Laboratory tests |
| Cardiovascular disorder, chest pain (new onset) (symptom/signs of) | 29 |  |  |  | Weak | Symptom/Sign |
| Neurological disorder, altered mental status (symptom/signs of) | 29 |  |  |  | Weak | Symptom/Sign |
| Neurological disorder, seizure (symptom/signs of) | 29 |  |  |  | Weak | Symptom/Sign |
| Respiratory disorder (symptoms/signs of) | 29 |  |  |  | Weak | Symptom/Sign |
| Glasgow Coma Scale, <15 (out of 15) [OR reference 15/15] |  | 16 | 22 |  | Weak | Vital signs |
| Alcohol abuse (history of) |  |  |  |  | Inconclusive | Comorbidities |
| Alcohol abuse (history of) |  |  |  |  | Inconclusive | Comorbidities |
| Alcohol consumption (history of) |  |  |  |  | Inconclusive | Comorbidities |
| Cardiovascular disorder, acute MI (history of) |  |  |  |  | Inconclusive | Comorbidities |
| Cardiovascular disorder, arrhythmia (history of) |  |  |  |  | Inconclusive | Comorbidities |
| Cardiovascular disorder, coronary artery disease (history of) |  |  |  |  | Inconclusive | Comorbidities |
| Cardiovascular disorder, hypertension (history of) |  |  |  |  | Inconclusive | Comorbidities |
| Gastrointestinal disorder, hepatic disease (severe) (history of) |  |  |  |  | Inconclusive | Comorbidities |
| Gastrointestinal disorder, hepatic dysfunction (history of) |  |  |  |  | Inconclusive | Comorbidities |
| Gastrointestinal disorder, hepatobiliary disease (history of) |  |  |  |  | Inconclusive | Comorbidities |
| Gastrointestinal disorder, peptic ulcer disease (history of) |  |  |  |  | Inconclusive | Comorbidities |
| Infective disorder, HIV (history of) |  |  |  |  | Inconclusive | Comorbidities |
| Infective disorder, immunocompromise (history of) |  |  |  |  | Inconclusive | Comorbidities |
| Infective disorder, immunocompromise (history of) |  |  |  |  | Inconclusive | Comorbidities |
| Malignancy (history of) |  |  |  |  | Inconclusive | Comorbidities |
| Malignancy, advanced (history of) |  |  |  |  | Inconclusive | Comorbidities |
| Metabolic disorder, diabetes type 2 (history of) |  |  |  |  | Inconclusive | Comorbidities |
| Metabolic disorder, endocrine disease (history of) |  |  |  |  | Inconclusive | Comorbidities |
| Neurological disorder, cerebrovascular disease (history of) |  |  |  |  | Inconclusive | Comorbidities |
| Neurological disorder, cerebrovascular disease (history of) |  |  |  |  | Inconclusive | Comorbidities |
| Neurological disorder, cerebrovascular disease  (history of) |  |  |  |  | Inconclusive | Comorbidities |
| Neurological disorder, dementia (history of) |  |  | 27 | 21 | Inconclusive | Comorbidities |
| Renal disorder, chronic kidney disease (history of) |  |  |  |  | Inconclusive | Comorbidities |
| Smoking status, Ex-Smoker (history of) |  |  |  |  | Inconclusive | Comorbidities |
| Age ≥ 80 years |  |  |  |  | Inconclusive | Demographic |
| Cardiovascular disorder (diagnosis of) |  |  |  |  | Inconclusive | Diagnosis |
| Cardiovascular disorder (diagnosis of) |  |  |  | 26 | Inconclusive | Diagnosis |
| Gastrointestinal disorder (diagnosis of) |  |  |  |  | Inconclusive | Diagnosis |
| Infective disorder (diagnosis of) |  |  | 26 |  | Inconclusive | Diagnosis |
| Malignancy (diagnosis of) |  |  |  |  | Inconclusive | Diagnosis |
| Metabolic disorder (diagnosis of) |  |  |  |  | Inconclusive | Diagnosis |
| Neurological disorder (diagnosis of) |  |  | 26 |  | Inconclusive | Diagnosis |
| Other Diagnosis |  |  | 26 |  | Inconclusive | Diagnosis |
| Respiratory disorder (diagnosis of) |  |  |  |  | Inconclusive | Diagnosis |
| Respiratory disorder, asthma (diagnosis of)[IRR reference no disease] |  |  |  |  | Inconclusive | Diagnosis |
| Albumin, mean (g/L) [lower in ICU arm][OR threshold ≤ 33.5] |  | 24 |  |  | Inconclusive | Laboratory tests |
| Bilirubin, median (umol/L) [higher in ICU arm][OR threshold ≥ 14.5] |  | 24 |  |  | Inconclusive | Laboratory tests |
| BNP, mean (ng/ml) |  |  |  |  | Inconclusive | Laboratory tests |
| Copeptin, increase in (pmol/L) |  | 25 |  |  | Inconclusive | Laboratory tests |
| Creatinine, median (mmol/L) [higher in ICU arm][OR threshold ≥ 105.5] |  | 24 |  |  | Inconclusive | Laboratory tests |
| D-dimer, mean (ng/ml) |  |  |  |  | Inconclusive | Laboratory tests |
| Haematocrit, mean (L/L) [lower in ICU arm][OR threshold ≤ 0.345] |  | 24 |  |  | Inconclusive | Laboratory tests |
| Haemoglobin, mean (g/L) [lower in ICU arm][OR threshold ≥ 106.5] |  | 24 |  |  | Inconclusive | Laboratory tests |
| pH, mean [lower in ICU arm][OR threshold ≤ 7.305] |  | 24 |  |  | Inconclusive | Laboratory tests |
| Pro-adrenomedullin, increase in (nmol/L) |  | 25 |  |  | Inconclusive | Laboratory tests |
| Red cell distribution width, increase in deciles (%) |  | 23 |  |  | Inconclusive | Laboratory tests |
| Total bicarbonate, mean (mmol/L) [lower in ICU arm] |  | 24 |  |  | Inconclusive | Laboratory tests |
| White cell count, < 12 (10x9/L) [higher % of in ICU arm] |  |  |  |  | Inconclusive | Laboratory tests |
| White cell count, <4 (10x9/L) [higher % of in ICU arm] |  |  | 27 |  | Inconclusive | Laboratory tests |
| Medication, Anti-Coagulant (patient taking) |  |  | 27 |  | Inconclusive | Medication |
| Medication, Anti-Platelet (patient taking) |  |  |  |  | Inconclusive | Medication |
| Medication, Corticosteriod (patient taking) |  |  |  |  | Inconclusive | Medication |
| Medication, Total count of (median) [higher in ICU arm] |  |  | 27 |  | Inconclusive | Medication |
| Deceleration Capacity, mean (m/s) (lower in ICU group) |  |  | 19 |  | Inconclusive | N/A |
| Infective disorder, fever (complaint of) |  |  |  |  | Inconclusive | Symptom/Sign |
| Neurological disorder (symptom of) |  |  | 26 |  | Inconclusive | Symptom/Sign |
| Other Presenting Complaint |  |  | 26 |  | Inconclusive | Symptom/Sign |
| Pain, except chest pain (symptom of) |  |  | 26 |  | Inconclusive | Symptom/Sign |
| Respiratory disorder (reason for RRS activation, symtom/signs of) |  |  |  |  | Inconclusive | Symptom/Sign |
| Respiratory disorder (symptom of) |  |  |  |  | Inconclusive | Symptom/Sign |
| Thoracic pain (symptom of) |  |  | 26 |  | Inconclusive | Symptom/Sign |
| Heart rate [<60 (bpm)] |  |  | 22 |  | Inconclusive | Vital signs |

^Both studies from Tsai et al (29)(30) come from the same patient data base. In accordance with the modified Grading System for Strength of Evidence, these two studies were only counted once (and weighted as a single high-quality study when shown together)
